# Alcohol Screening, Brief Intervention, and Referral to Treatment (SBIRT) for Girls and Women

**DOI:** 10.35946/arcr.v40.2.07

**Published:** 2020-08-13

**Authors:** Kyndal Hammock, Mary M. Velasquez, Hanan Alwan, Kirk von Sternberg

**Affiliations:** 1 Health Behavior Research and Training Institute, University of Texas at Austin, Austin, Texas

**Keywords:** brief intervention, risk, alcohol, SBIRT, screening, women, female adolescents

## Abstract

Females ages 12 and older are the fastest growing segment of alcohol consumers in the United States, with the past decade showing a 16% increase in alcohol use per 12-month period and a 58% increase in high-risk drinking (i.e., > 3 drinks in a day and/or > 7 drinks in a week) per 12-month period. The increase in alcohol use and risk drinking poses unique and serious consequences for women. Women have a more rapid progression to alcohol-related problems and alcohol use disorders (AUD) than men, and if pregnant, women can potentially expose the fetus to alcohol. Screening, brief intervention, and referral to treatment (SBIRT) is an evidence-based, integrated public health approach used to identify and address risky alcohol use among women in a variety of health and social service settings. This article presents the current status of SBIRT among girls ages 12 and older, women of childbearing age, and older women. Screening instruments, brief interventions, and implementation issues specific to women of all ages are described. Through this review of the current literature, care providers can determine best practices for the prevention and treatment of risk drinking in women of all ages presenting in health care settings.

## INTRODUCTION

Alcohol is the most commonly consumed substance among Americans ages 12 and older, and women are the fastest growing segment of alcohol consumers in the United States.^1^,^2^ Female alcohol consumption that meets criteria for risk drinking, defined as more than three drinks in a single day or more than seven drinks per week, has the potential to negatively affect the health and well-being of women across their life spans.^3^ Evidence indicates converging patterns of alcohol consumption between men and women resulting from recent increases in female alcohol use behaviors.^2^,^4^,^5^ For instance, data collected in the past decade reveal that among U.S. women, alcohol use increased by 16% per 12-month period, high-risk drinking increased by 58% per 12-month period, and diagnoses of alcohol use disorder (AUD)—as defined in the fourth edition of the *Diagnostic and Statistical Manual of Mental Disorders*—increased by 84% per 12-month period.^2^ These increases have unique and serious consequences for women given that they experience a more rapid progression—at lower consumption levels—to alcohol-related problems and AUD than men.^6^,^7^

This recent increase in female alcohol consumption underlines a need for additional research and clinical efforts to address alcohol use among girls and women.^2^,^4^ Because risky drinking poses unique and detrimental consequences to all women, age and life circumstances should not preclude any subset of girls or women from research or clinical efforts to address this growing public health concern. Indeed, risky alcohol use is prevalent among young girls;^8^,^9^ pregnant and postpartum women;^10^,^11^ victims of child abuse,^12^ sexual trauma,^13^ and intimate partner violence;^14^ female veterans;^15^ incarcerated girls and women;^16^ sexual-minority women;^17^ and older women.^5^ Due to alcohol’s nondiscriminatory nature towards varying groups of women, universal screening, brief intervention, and referral to treatment (SBIRT) appears to be an appropriate, evidence-based public health approach capable of identifying and addressing risky alcohol use among females in a variety of health and social service settings.^18^ This article presents a review of the literature regarding the role of SBIRT in addressing risky alcohol consumption among girls (ages 12 to 18), women of childbearing age (i.e., ages 18 to 44), and older women (i.e., ages 65 and older). There is a general lack of currently available research data specific to women ages 45 to 64, but other than risk of pregnancy associated with women ages 18 to 44, the role of SBIRT is similar for women ages 45 to 64 to that for younger women. Databases used for this review include PubMed, Cochrane Library, Google Scholar, and Academic Search Complete. The reference lists of selected articles and texts were also explored.

## SBIRT

The current SBIRT model is based on a recommendation from the National Academy of Medicine (previously called the Institute of Medicine) to develop integrated service systems that bridge the gap between primary prevention and treatment services for individuals with problematic alcohol and/or illicit drug use.^19^ In 2003, the Substance Abuse and Mental Health Services Administration (SAMHSA) established an initial SBIRT grant program, with the intent of integrating behavioral health services into settings where individuals who engaged in risky substance use behaviors could be identified and offered an appropriate level of intervention and care.^20^ Findings from this initiative suggest that SBIRT is associated with improvements in alcohol use outcomes.^20^,^21^

The U.S. Preventive Services Task Force (USPSTF), an independent entity consisting of experts in preventive medicine, recently updated its recommendation for care providers. This update recommends that care providers screen all adults ages 18 and older, including pregnant women, for risky alcohol use and provide brief behavioral counseling interventions, when appropriate, to reduce unhealthy alcohol use.^22^ Screening adolescents younger than age 18 was not included in the updated recommendation; the USPSTF concluded that there is insufficient evidence to properly assess the benefits versus risks for alcohol screening and brief interventions (BI).^22^ The American Academy of Pediatrics (AAP), however, has recommended the practice of screening and providing BI to adolescent alcohol users, citing low cost, minimal potential for harm, and emerging evidence of the benefit that SBIRT may have among adolescent alcohol users.^23^

SBIRT is intended to identify, reduce, and prevent problematic alcohol use behaviors and is made up of three key components: screening, brief intervention, and referral to treatment. Ideally, the first step of the SBIRT process is to administer a validated prescreen instrument to all presenting individuals in a practice setting, as part of the routine intake procedure, to identify those who are drinking at or above risky levels.^24^,^25^,^26^ When prescreen instruments detect consumption at risk levels, measured by standard drinks (14 grams or 0.6 fluid ounces of pure alcohol) consumed, a more comprehensive assessment can be conducted to gauge the severity of alcohol use and inform BI and/or treatment options.^3^ For example, the National Council for Behavioral Health recommends that a symptom checklist or other validated assessment be used to obtain alcohol-related symptoms from individuals whose prescreen indicates risky consumption.^26^ If it is determined that an individual is consuming alcohol at moderate risk levels (i.e., above NIAAA threshold for low-risk consumption but not at a level indicative of AUD), then the second step in the SBIRT process is to complete a BI protocol. BIs are often based on principles of motivational interviewing (MI) and aim to increase awareness of alcohol-related risks and consequences and to encourage motivation for change. If an individual is identified to be drinking at levels that are suggestive of AUD, then referral to specialized treatment for further assessment and care is recommended.^27^

## SCREENING

SBIRT begins with universal screening, the goal of which is to identify individuals who have, or are at risk of developing, alcohol-related problems.^27^ Universal screening that is adherent to SBIRT standards, and described in multiple SBIRT practice guides, involves the administration of a validated prescreen instrument that has been limited to a few questions needing only simple responses.^24^,^26^,^28^,^29^ Ideal screening instruments have high sensitivity and specificity ratings, with cutoff scores designed to maximize both ratings in order to minimize false positives and false negatives.^30^ However, for prescreen instruments that are intended to be universally administered, priority is often given to sensitivity over specificity so that individuals in large clinical populations (e.g., women in primary or reproductive care settings who consume alcohol while pregnant) are appropriately identified for further assessment.^30^,^31^

This article classifies screening instruments into prescreen and screen categories. The purpose of prescreening is to assess an individual’s frequency and quantity of alcohol use to determine whether the person is drinking at age-specific risk levels, whereas the purpose of screening is to elicit alcohol-related symptoms from those that have been identified as drinking at risk levels. Prescreens and screens should work in succession, and because many instruments are capable of serving both screening purposes, this dual process is sometimes consolidated into a single step within clinical practice settings.

Universal prescreening and screening efforts must be conducted using valid, age-appropriate instruments with cutoff scores that are tailored to a population’s sex and age (see Table 1).^32^ Following is an overview of screening practices and instruments that have been validated for use within specified age groups of girls and women.

### Adolescents

NIAAA, SAMHSA, and AAP recommend that care providers screen all adolescents and young adults ages 12 to 21 for alcohol and substance use behaviors using validated screening instruments on a yearly basis and, as needed, during acute care visits.^33^ There are currently three prescreen options that are applicable to adolescents: the two age-specific questions found in NIAAA’s *Alcohol Screening and Brief Intervention for Youth: A Practitioner’s Guide*;^29^ the first three questions of the Screening to Brief Intervention (S2BI); and the three-item Alcohol Use Disorders Identification Test–Concise (AUDIT-C).^33^ The two age-specific questions found within NIAAA’s guide ask about an adolescent’s personal alcohol use as well as that of their friends and is appropriate for children and adolescents between the ages of 9 and 18. This AAP-endorsed guide includes elementary, middle, and high school age-appropriate variations of these two questions, which allow for accurate correlation of patient responses to current or potential risky alcohol consumption.^29^ The S2BI instrument screens for alcohol, tobacco, marijuana, and illicit drug use by asking a single frequency-of-use question per substance. This screener is highly sensitive and specific at discerning among various risk categories, from no use to severe substance use disorder (SUD). Although not a formal diagnostic instrument, the S2BI has been shown to closely correspond with the likelihood of current SUD.^34^ The AUDIT-C, validated for use with young people ages 12 to 19, has three questions to identify the quantity and frequency of alcohol consumption.^32^,^35^,^36^

When adolescents score positive on a prescreen instrument, indicating some level of risky alcohol consumption, they are asked to respond to additional, more specific screening questions to determine whether a BI or referral to treatment is appropriate. Screening instruments that have been validated for use with adolescents and can be used to inform next steps include the 10-item Alcohol Use Disorders Identification Test (AUDIT); the Brief Screener for Tobacco, Alcohol, and Other Drugs (BSTAD); and the Car, Relax, Alone, Forget, Friends, Trouble (CRAFFT) screening instrument.^23^,^32^,^37^ The AUDIT is the most widely tested alcohol screening instrument and is commonly used to assist in the early identification of individuals engaging in risky drinking behaviors.^22^ Furthermore, the AUDIT has been validated for use among young people,and evidence suggests a lack of gender bias between female and male adolescents.^32^,^35^ The BSTAD, an adaptation of the questions found within NIAAA’s guide includes questions on alcohol, tobacco, and drugs, and has been shown to be highly sensitive and specific at identifying risky past-year alcohol use among adolescents ages 12 to 17.^38^ Recommended by both NIAAA and AAP, the CRAFFT has been validated across pediatric settings to identify risky substance use behaviors among adolescents.^18^,^39^ Interestingly, the CRAFFT was able to detect preconception substance use in a small cohort of pregnant adolescents and young women between ages 17 and 25.^33^,^40^ The CRAFFT has many advantages, including a short administration time and high sensitivity and specificity.^33^ It also shows no evidence of gender bias.^36^

Screening adolescents for risky alcohol use can be incorporated into psychosocial approaches. For example, the home environment, education and employment, eating, peer-related activities, drugs, sexuality, suicide/depression, and safety from injury and violence (HEEADSSS) and the strengths, school, home, activities, drugs/substance use, emotions/depression, sexuality, safety (SSHADESS) tools are interview frameworks specifically designed for use with adolescents in health care settings.^23^,^33^ The HEEADSSS interview is a practical, complementary strategy that establishes rapport by asking less threatening questions at the beginning of the encounter before transitioning to more personal or potentially intrusive topics such as substance use.^33^ The SSHADESS interview covers the same life areas as the HEEADSSS, but it also underscores adolescents’ resiliency by identifying their perceived and realized strengths before asking questions related to environmental context or risky behaviors.^23^

A caveat is that an assurance of confidentiality is needed to improve the accuracy of adolescent screening responses. Because most adolescents are not comfortable discussing topics like alcohol use and sexual activity in the presence of a parent or guardian, clinicians are encouraged to create scripts or other procedures to excuse the accompanying adult from a portion of the health exam.^33^ For example, asking the adult to leave the room during the physical exam portion validates the adolescent’s developmental need for privacy and creates space for a confidential discussion concerning alcohol and other potentially risky behaviors.^33^ Federal and state privacy laws entitle adolescents to privacy regarding substance use treatment, so adolescents may further benefit from a script ensuring that what is disclosed to the provider will not be shared with their caregiver unless an immediate risk of injury to oneself or another is divulged.^33^

### Women of Childbearing Age

For women of childbearing age, the USPSTF supports the use of brief prescreening instruments for alcohol with 1 to 3 items—such as the AUDIT-C or the NIAAA-recommended Single Alcohol Screening Question (SASQ), also referred to as the “single binge drinking question”—to quickly identify women who may be at risk.^22^,^41^,^42^ The use of a single binge drinking question has also been recommended as a first step to effectively and efficiently identify women who are likely to be at risk of an alcohol-exposed pregnancy (AEP).^43^ For example, a single binge drinking question was found to correctly identify 99% of women, from two countries and cultures, who had been identified as at risk of an AEP.^43^ The Quick Drinking Screen (QDS) is another brief instrument that is efficacious at initially identifying women at risk of an AEP.^44^ Items from the QDS were measured against data collected from a 90-day timeline followback (TLFB) assessment among a sample of women already determined to be at risk of an AEP. The results found that the women’s answers to QDS items were highly similar to their 90-day TLFB responses.^43^

Once a brief prescreening measure identifies a woman who is likely to be at risk for alcohol misuse and/or an AEP, it is recommended that a more comprehensive instrument be administered.^22^,^43^ For example, the 10-item AUDIT is an efficacious measure that has been validated for use with this population.^45^ There are also several assessments designed specifically for women of childbearing age, including pregnant women and women at risk of an AEP. It is recommended that universal prescreening among women of childbearing age be used to identify and assess women at risk of an AEP.^45^,^46^ Screening this population provides the opportunity for early intervention among women who may have consumed alcohol prior to becoming aware of their pregnancy. Screening also alerts care providers of consumption levels indicative of AUD so that they can refer these women for specialized treatment.

The Tolerance, Annoyed, Cut Down, Eye-Opener (T-ACE) questionnaire was the first validated screening instrument developed to identify drinking among pregnant women. It is often used in reproductive settings, including maternity care and gynecological clinics.^25^,^31^ In comparison to the AUDIT, the four-item T-ACE has shown slightly higher sensitivity at detecting current alcohol consumption among pregnant women.^31^ In addition, the T-ACE accurately identifies varying levels of alcohol consumption and is acceptable for use among culturally diverse obstetric populations.^31^ The five-item Tolerance, Worried, Eye-Opener, Amnesia, K/Cut Down (TWEAK) screening instrument is another validated questionnaire for identifying drinking among women, including those who are pregnant and those at risk of an AEP.^25^,^31^,^45^ Although the TWEAK questionnaire appears to be highly sensitive at identifying heavy patterns of alcohol consumption, primarily among white women, it is less sensitive at detecting lower levels of drinking that could still be considered at risk.^25^,^47^

In addition to the T-ACE and TWEAK, the USPSTF also recommends the Normal Drinker, Eye-Opener, Tolerance (NET), and the Parents, Partner, Past, Present Pregnancy (4P’s Plus) as screening measures capable of assessing alcohol use among pregnant women.^22^,^47^,^48^ Nonetheless, the T-ACE and TWEAK reportedly perform best among pregnant women and do not appear to have a significant advantage over one another, because both are well-validated screening measures that can be quickly administered in a variety of women’s health settings.^18^

### Older Women

Older women are often missed by screening efforts because their alcohol-related symptoms are often mistaken for signs of aging.^49^ For this reason, systems must be put into place to ensure universal screening on a recurring basis in settings that care for older women.^50^ Alcohol screening should take place any time new mental or physical health symptoms arise, before prescribing a new medication, in response to major life changes (e.g., retirement, death of a spouse), and on a yearly basis as part of routine physical and mental health services.^50^,^51^ Providers should be aware that a history of risky alcohol use among older adults often predicts future increases in drinking.^50^ Prescreening questions like “During your lifetime, have you ever used alcohol?” followed by “During the past year, have you had four or more drinks on a single occasion?” help to determine whether more comprehensive assessments are warranted.^51^,^52^ The AUDIT-C and the two-item Substance Use Brief Screen (SUBS) are also prescreen options available for use with this population.^53^–^55^

Several screening instruments have been validated for use with older adults. Measures like the AUDIT include screening questions on lifetime problems to assess current alcohol-related risk.^54^,^56^ Other screening tools include the Cut Down, Annoyed, Guilty, Eye-Opener (CAGE), the Michigan Alcoholism Screening Test—Geriatric Version (MAST-G), the Short MAST-G, and the Comorbidity Alcohol Risk Evaluation Tool (CARET).^54^,^57^ All of these instruments gather information about the level of consumption and offer decision support for care providers.^50^,^54^ In general, alcohol screening and assessment instruments among older women should contain questions about the frequency and quantity of alcohol use, experiences with drinking-related consequences, medication use, and feelings of depression.^50^

## SCREENING RECOMMENDATIONS

There are very few studies on alcohol screening specific to adolescent females and older adult females beyond childbearing age, with a majority of information coming from mixed-gender studies. The largest body of evidence on screening women is for those of childbearing age, likely due to the added risks and harms associated with prenatal alcohol exposure. Nonetheless, universal screening should begin in early adolescence and be repeated at regular intervals across settings that provide health care and social services to girls and women. However, although alcohol screening instruments elicit important information about an individual’s level of risk and alcohol-related symptoms, these tools are not a replacement for a complete substance use assessment. Because these instruments are brief and, in many cases, can be self-administered, it is often recommended that care providers use them as decision support aids to guide additional steps based on the preliminary level of risk indicated by these alcohol screening instruments.

The successful implementation of a screening protocol depends on the setting in which it is delivered. For example, settings with access to interdisciplinary professionals may find that longer, more thorough assessment instruments are practical, whereas settings with fewer resources are likely to benefit from utilizing brief instruments like the AUDIT, which has been validated for use across age groups.^32^,^35^,^56^ Additionally, questions or measures may be added to assessment protocols to identify other factors known to be associated with female alcohol use behaviors (e.g., age of onset, depression and anxiety, childhood and/or intimate partner abuse, co-occurring substance use behaviors) to better inform BI and referral to treatment practices.^13^,^16^,^58^,^59^ Moreover, care providers need to remain mindful regarding the language they use to describe alcohol-related concerns so as not to further stigmatize female populations.^60^ For example, some women may be sensitive to language such as “alcoholic,” “addict,” or “abuser”; the use of such language may dissuade women from providing relevant information pertaining to their alcohol use behaviors. Therefore, care providers are advised to use medically accurate terms throughout their discussions regarding alcohol and substance use behaviors.^55^,^60^

## BRIEF INTERVENTIONS

BIs are evidence-based practices that are short, targeted conversations between women and clinicians that follow screening results indicative of risky alcohol consumption. The overall goal of BIs is to help adolescent girls and women who are at risk of alcohol-related consequences by increasing their awareness about the ways alcohol use may put them at risk and encouraging their self-motivation for change.^27^,^61^ Common components of BIs include conversations on standard drink sizes, low- versus high-risk drinking limits, and potential health effects and social consequences of drinking.^3^,^62^ Another common element of BIs is providing personalized normative feedback, with evidence supporting the use of gender-specific feedback for women.^63^,^64^,^65^ BIs can be delivered by professionals with different backgrounds and expertise, and they can take place in face-to-face settings, over the phone, or through electronic means.^61^,^66^ How effective BIs are can depend on the number of sessions and length of time allotted for each session. For example, systematic reviews and meta-analyses have found that very brief (i.e., ≤ 5 min) and brief single-contact interventions (i.e., 6 to 15 min) tend to be less effective than brief multicontact interventions (i.e., each contact ≤ 15 min), which evidence shows is the most effective across populations and outcomes.^18^,^63^,^67^ Additionally, one meta-analysis found that extended BIs (defined by the author as BIs that required several visits, or multicontact interventions) resulted in significant change in alcohol consumption for women but not men.^68^

BIs for risky alcohol use are often based on the principles of MI. Using this collaborative, client-centered approach, providers help females explore and resolve their ambivalence toward changing unhealthy behaviors (e.g., alcohol consumption at risk levels).^69^ A core tenet of MI is the use of nonconfrontational techniques to allow individuals to guide themselves toward change without feeling the need to defend their choices.^69^

### Adolescents

AAP recommends basing the degree of intervention delivery for youth on the level of risk identified at the time of screening. When no alcohol use is reported, clinicians are encouraged to provide positive verbal reinforcements to motivate continued abstinence. Evidence suggests that even a few positive words from a health care provider may delay alcohol use initiation, and thus extend time for adolescent brain maturation.^23^ These positive reinforcements may be critical for female adolescents to receive, especially girls at risk of early alcohol initiation,^7^,^58^ because of the detrimental effects of alcohol on the female developing brain.^70^ When infrequent alcohol use is endorsed by female adolescents, such as when an S2BI result indicates alcohol use of one to two times the previous year, it is recommended that care providers advise adolescents to abstain. This advice may combine information on negative health consequences with recognition of personal strengths and positive attributes.^23^

BIs are recommended when an adolescent screens positive for drinking at risky levels. Evidence from a recent meta-analysis of 185 studies examining the effects of alcohol-related BIs for adolescents and young adults found that the interventions effectively reduced drinking and alcohol-related consequences, with effects lasting up to 1 year and showing no demographic variance.^65^

BIs that utilize MI have been found to be effective with substance-using adolescent populations. Much of the research supporting this view falls into the harm-reduction continuum: that is, adolescents do not move directly into abstinence but rather gradually decrease their risky behavior.^71^,^72^ In addition to the effectiveness of MI techniques within this population, a systematic review and meta-analysis conducted by Carney and Myers also found that adolescents showed a preference for individualized interventions (i.e., compared with a group format) conducted over multiple sessions (i.e., compared with a single event).^67^

In alignment with the USPSTF finding of there being insufficient evidence to evaluate the utility of BIs among alcohol-using adolescent populations, evidence specific to adolescent females who receive brief alcohol interventions is also lacking and warrants future investigation. In a recent systematic review and meta-analysis of the literature on brief alcohol interventions for adolescents and young adults, Tanner-Smith and Lipsey found a limited number of studies with boy-only or girl-only samples that reported little to no evidence of differential effectiveness based on gender.^65^ There is some evidence, however, suggesting that BIs for alcohol use may be particularly effective for adolescent girls, especially when the provider is also female and the information is delivered in the context of an ongoing provider–patient relationship.^73^

### Women of Childbearing Age

There is strong evidence supporting the use of BIs among pregnant and nonpregnant women of childbearing age as a means of reducing levels of alcohol consumption and risks associated with AEPs.^18^,^62^,^74^ For example, in one large multisite trial, approximately 69% of women who, at intake, were drinking at risky levels and not using effective contraceptive methods reduced their risk of an AEP at the 9-month follow-up after receiving an intervention incorporating MI. The women in this study achieved risk reduction by abstaining from alcohol or drinking below risky levels, by using effective contraceptive methods every time they had vaginal intercourse with a fertile male, or both.^75^ A number of randomized controlled trials with pregnant women have also reported significant reductions in alcohol use and improved newborn outcomes following the facilitation of BIs.^62^

In addition to previously mentioned common components of BIs (e.g., personalized normative feedback), interventions with women of childbearing age often also include feedback on the potential effects of alcohol on fetal and child development.^25^,^64^ It is recommended that postpartum women receive information on infant exposure to alcohol through breastmilk and that contraceptive use should be incorporated into BIs with nonpregnant women who are at risk of an AEP.^25^,^64^

Efficacious prevention and intervention programs have been developed for use with women of childbearing age. One example is the CHOICES program and its adaptations: BALANCE, EARLY, and CHOICES Plus.^76^,^77^,^78^ CHOICES is an established AEP prevention program based on the principles of MI and designed to provide nonpregnant women of childbearing age with information to help them make informed choices on ways to avoid an AEP.^43^ The CHOICES protocol has been widely disseminated across health and social service settings (e.g., primary care facilities, jails, sexually transmitted disease clinics).^75^,^78^,^79^ Also, as a result of meeting rigorous peer-review criteria, the CHOICES program was included in SAMHSA’s Evidence-Based Practices Resource Center (https://www.cdc.gov/ncbddd/fasd/choices-importance-preventing-alcohol-exposed-pregnancies.html).

### Older Women

Although limited, studies on BIs with older adults suggest that BIs are effective at reducing risky alcohol consumption, with sustained reductions ranging from 2 to 18 months.^80^,^81^,^82^ The content and format of most BIs are similar, as are the recommendations, whether delivered to younger or older cohorts. For example, providers are advised to use nonstigmatizing and nonjudgmental language when discussing screening results and any potential alcohol-related health consequences with women.^55^ Regarding older women, some experts suggest that providers may find that incorporating the women’s family and friends into various parts of the BI process may prove successful.^51^

### Other BIs

Multiple BI models have been created to aid in the facilitation of BI conversations.^25^,^27^ A systematic review of BIs for risky drinking in primary care settings reported that a majority are arranged according to the SAMHSA-endorsed Feedback, Responsibility, Advice, Menu of strategies, Empathy, Self-efficacy (FRAMES) model.^33^,^64^ Other BI models that are endorsed by SAMHSA include the Feedback, Listen, Options (FLO) model, the Brief Negotiated Interview (BNI) Steps, and the BNI and Active Referral to Treatment: Provider Training Algorithms.^27^ All of these models serve as useful guides for delivering BIs and are presumed to be equally efficacious regardless of age or gender. Practitioners should choose the model that best suits their work setting.

In summary, BIs are valuable tools for reducing alcohol consumption and its associated risks (e.g., AEPs). It is vital to consider that despite a number of randomized controlled trials suggesting similar efficacy for brief alcohol interventions among women and men,^83^,^84^ women have been less likely to receive BIs in practice. As such, lending attention to this issue is critical considering that the prevalence rates for alcohol use among women are rising.^85^

## REFERRAL TO TREATMENT

Referral to treatment is a process designed to assist women with accessing specialized treatment, selecting facilities, and navigating barriers that may prevent treatment engagement.^27^ Treatment options for women with AUD may include residential treatment, outpatient psychological therapy (e.g., family, group, conjoint, individual), medication-assisted treatment, self-help or support group programs (e.g., 12-step programs such as Alcoholics Anonymous), harm reduction approaches, use of a recovery coach, or any combination of these. There are also treatment options that cater exclusively to women, such as the Women for Sobriety program and women-only Alcoholics Anonymous groups. Specialized alcohol treatment should be personalized to the woman, taking into account her medical, social, and cultural needs. Providers should be aware of local treatment options in order to conduct warm handoffs—referrals facilitated in the presence of the patient to encourage communication and partnership between the patient and treatment team—when needed. Providers should also pay special attention to the treatment selection for pregnant and postpartum women to ensure that appropriate medical care and social support options are available.^25^ Providers may also choose to access SAMHSA’s online resource guide, which includes samples of scripts, procedures, and links to treatment locator websites.^27^ Other referral resources include NIAAA’s online Alcohol Treatment Navigator tool (https://alcoholtreatment.niaaa.nih.gov) and NIAAA’s publicly available resource guides, with information specific to referrals: *Alcohol Screening and Brief Intervention for Youth: A Practitioner’s Guide*^29^ and *Helping Patients Who Drink Too Much: A Clinician’s Guide*.^28^

Referral to treatment is a critical, yet often overlooked, component of SBIRT. Although some studies have found it effective to link individuals to specialty treatments,^86^,^87^ evidence from many others suggests that it is often difficult to link individuals in need of alcohol-related specialized care to substance use treatment services. For example, a meta-analysis of nine studies found no evidence that brief alcohol interventions were efficacious for increasing the use of alcohol-related services.^88^ Referral to treatment is further compounded by gender-specific barriers to treatment that impact women’s ability to engage in services. In general, women are less likely than men to initiate alcohol treatment services, and when they do, research suggests that women often contend with stigma, negative staff attitudes, lack of affordable or safe childcare options, and concerns over child custody.^89^ When they do access treatment services, more women than men present with histories of trauma and abuse, psychological distress and mental health concerns, interpersonal and family-related issues, and financial constraints.^90^ Barriers on a systemic level include lack of treatment options because of geographic isolation and lack of awareness among care providers regarding local treatment options that are capable of addressing the unique needs of adolescent girls and women in treatment settings.^89^

## BARRIERS AND FACILITATORS TO SBIRT IMPLEMENTATION

A number of health and social service providers (e.g., physicians, nurses, social workers, psychologists, midwives) are qualified to effectively implement SBIRT across a variety of patient and client settings. However, studies of SBIRT implementation reveal that few providers feel comfortable doing so, with the lowest screening and counseling rates seen among young adult and women’s reproductive care providers.^18^ For example, one study found that one-third of women who endorsed alcohol consumption in women’s health clinics were not asked how much they drank and that a majority of women drinking at risk levels did not receive advice on low-risk limits.^91^ Another study concluded that approximately half of women at risk of an AEP did not receive information pertaining to this risk from their health care providers.^91^ These findings corroborate national survey data of family planning clinicians, which found that of these clinicians, approximately one-third used a validated screening measure and one-fifth provided a referral that consisted of more than a list of treatment options.^92^

Qualitative analyses conducted among health care providers have revealed several common barriers to implementing SBIRT, including time constraints, competing priorities, cost, and privacy and confidentiality concerns.^93^–^96^ Barriers that pediatric providers cited include concerns regarding the willingness of adolescents to return for follow-up, limited access to and knowledge of adolescent treatment programs or local expertise, and confidentiality concerns.^94^ Additional SBIRT barriers that prenatal care providers identified included lack of rapport between providers and women presenting for an initial prenatal consultation; providers’ misperception that there is a low prevalence of alcohol use by pregnant women; providers’ lack of skills, training, and follow-up protocol; women’s underreporting or false disclosure of alcohol consumption; and providers’ concerns over creating guilt and anxiety among pregnant women.^95^,^96^

Many of these provider-identified barriers should be considered in combination with, and resulting from, U.S. state policies mandating that health care providers report perinatal substance use to child welfare agencies.^97^,^98^ For instance, in 2017, Jarlenski and colleagues conducted a systematic content analysis that identified 24 states with statutes around reporting perinatal substance use by health care providers. Twenty of the states identified had mandatory reporting statutes, while 11 states specified a penalty capable of resulting in a misdemeanor charge for health care providers who failed to report known perinatal substance use.^98^ Furthermore, some state statutes allow for involuntary commitment and custody loss solely as a result of prenatal substance use, thus creating an ethical and moral dilemma for prenatal care providers because this violates the principles of patient autonomy and beneficence.^99^ This issue was further complicated for prenatal care providers by updated recommendations from the American College of Obstetricians and Gynecologists and the Centers for Disease Control and Prevention, which advise providers to conduct universal screening at initial prenatal appointments.^46^,^98^

In addition to the barriers faced by prenatal care providers, pregnant women engaged in substance use behaviors often face their own barriers to receiving care, such as fear of stigmatization and legal consequences. This may result in a lack of engagement in prenatal care altogether, thus eliminating the potential for SBIRT implementation and posing significant risks to the health of both mother and child.^60^

Older women also face unique barriers to alcohol intervention and treatment efforts. These include financial limitations and lack of mobility and transportation. Older women also report higher rates of stigma, shame, and guilt than younger women, which may lead to an increased prevalence of isolation, anxiety, and depression.^51^

### Approaches to Facilitating SBIRT Implementation

In response to the many recognized barriers, research has begun to identify approaches that facilitate successful SBIRT implementation. So far, evidence suggests that having a practice champion, utilizing an interprofessional team, communicating the details of each SBIRT step, developing relationships with referral partners, instituting ongoing SBIRT training for sustainability, aligning SBIRT practices with the organization’s flow, and integrating SBIRT into electronic health records are all ways to facilitate ongoing SBIRT efforts.^24^ Additionally, a study of ongoing SBIRT facilitation compared usual care and two adolescent SBIRT delivery modalities (pediatrician-only and pediatrician with an embedded behavioral clinician) and found that although substance use outcomes did not differ between pediatrician-only and embedded behavioral clinician groups, adolescents in the embedded group reported fewer depression symptoms at follow-up.^100^ The inclusion of a behavioral clinician in pediatric settings may be especially beneficial to adolescent girls in light of recent evidence that higher levels of depression severity among girls ages 13 to 16 predicted alcohol use in the following year.^59^

### Technology

The use of technology is an additional option for overcoming SBIRT barriers in clinical settings that lack available staff and time resources for ongoing face-to-face implementation.^101^ Technology is increasingly being used to facilitate various SBIRT components, with preliminary evidence observed among adolescent girls and women looking promising.^74^,^102^,^103^ A recent systematic review of women’s experiences with technology-based screening found that the perception of anonymity made it easier to divulge potentially stigmatizing information compared to in-person, face-to-face screening methods. Therefore, technology-based screening has the potential to increase disclosure rates and intervention receipt.^104^ Studies also suggest that women feel less embarrassed and less afraid of judgment when they participate in technology-based interventions, and the flexibility offered by some technology-based treatments may also be appealing to women who are not willing or able to participate in more formal treatment programs because of family and societal roles.^104^

Nevertheless, whether electronic SBIRT can be effective as a stand-alone entity has yet to be established. One recent study demonstrated successful implementation of a technology-based alcohol intervention (i.e., sans personnel) among women of childbearing age;^66^ however, interaction findings from other studies suggest that various female groups may have other intervention needs.^105^ For example, Choo and colleagues reported that although female victims of intimate partner violence were receptive to electronic screening and advice, they also desired empathy and compassion from human interaction provided during intervention delivery.^105^ Still, evidence has suggested that electronically delivered SBIRT components are mutually beneficial to both women and providers.^103^,^106^ In the future, the use of electronic approaches could also assist in the translation of research findings into routine care settings by standardizing intervention delivery methods while maintaining wide applicability across health and social service settings.^107^

## FUTURE DIRECTIONS

More research is needed to evaluate the effectiveness, efficacy, and feasibility of SBIRT practices among females, primarily those in younger and older cohorts, and those at risk of AEPs.^4^,^10^,^59^,^64^ Recent reports showed increases in alcohol use among adolescent girls, with evidence suggesting a reversal from traditional male excess to slight female excess in 8th grade, and by 12th grade, 35% of girls reported past-month alcohol use, corresponding to a 250% increase from 8th grade.^9^,^102^ Age of alcohol use initiation is particularly worrisome among adolescent females, given that early initiating females drink more than all male adolescents from ages 12 to 17.^8^ Additionally, the association between depression severity and alcohol use appears to be more salient for early adolescent girls than for boys of the same age, with observations suggesting that alcohol use both predicts and is a consequence of depression.^59^ Research is also needed to address alcohol use among older women due to population increases. Given the aging of the baby-boom generation, population projections estimate that by 2040, the proportion of women to men ages 65 or older will be 127 to 100.^51^,^108^

SBIRT is essential for the ongoing identification and intervention of risky alcohol use behaviors among adolescent girls and women. As the prevalence rate of female alcohol use increases, so too should the implementation of SBIRT. These prevention and intervention efforts can help promote lifelong health and well-being among women, with special attention paid to younger and older cohorts, and those at risk of an AEP.

## Figures and Tables

**Table 1:** Alcohol Screening Instruments

Instrument	No. of Items in Instrument	Approx. Time to Administer (min)	Applicable Population	Scoring That Indicates Risk and Statistical Performance (Sensitivity; Specificity)	Copyright, Source(s), and Cost‡‡	Link(s)
NIAAA *Alcohol Screening and Brief Intervention for Youth: A Practitioner’s Guide*^29^	2 to 3 depending on severity	~2	Adolescents ages 9 to 18	Elementary or middle school adolescents (≤ 15 years old) reporting any alcohol use (0.89; 0.91)^33^ High school adolescents (≥ 16 years old) reporting ≥ 6 days of past-year alcohol use (0.88; 0.81)^33^	Copyright: N/A Source: N/A Cost: Free online	Publicly available NIAAA guide containing screening questions (page 8): https://www.niaaa.nih.gov/sites/default/files/publications/YouthGuide.pdf
Screening to Brief Intervention (S2BI)^34^*	3 (additional 4 if past-year use indicated)	~2	Adolescents ages 12 to 17	Adolescents reporting alcohol use o*nce or twice* in the past year (0.96; 0.92) Adolescents reporting alcohol use *monthly* in the past year (0.79; 0.96) Adolescents reporting alcohol use *weekly or more* in the past year (1.00; 0.88)	Copyright: N/A Source: N/A Cost: Free online	Publicly available NIDA link to online version with options for patient or clinician administration: https://www.drugabuse.gov/ast/s2bi/#/
Brief Screener for Tobacco, Alcohol, and Other Drugs (BSTAD)^38^*	6 (additional 3 to 11 if past-year use indicated)	~2	Adolescents ages 12 to 17	≥ 2 days of past-year alcohol use (0.96; 0.85)	Copyright: N/A Source: N/A Cost: Free online	Publicly available NIDA link to web-based instrument with options for patient or clinician administration: https://www.drugabuse.gov/ast/bstad/#/
Alcohol Use Disorders Identification Test (AUDIT)	10	~2 to 3	Adolescent girls ages 12 to 19, adults,§ pregnant women, older adults	Positive score indicating risk: Adolescent girls: ≥ 5 (0.95; 0.77)^32^ Adults: ≥ 8 (0.38–0.73; 0.89–0.97)^18^** Pregnant women: > 0^18^ Older adults: ≥ 5 (0.86; 0.87)^54^	Copyright: 1989, Thomas Babor and the World Health Organization Sources: World Health Organization, Division of Mental Health & Prevention of Substance Abuse, 1211 Geneva 27, Switzerland Email: Publications@who.int Thomas F. Babor, Alcohol Research Center, University of Connecticut, Farmington, CT Cost: Core questionnaire can be reproduced without permission; test and manual are free; training module costs $75	Publicly available link to self-report instrument: https://cde.drugabuse.gov/sites/nida_cde/files/AUDIT-SelfReport_v1.0_2014May20.pdf
Alcohol Use Disorders Identification Test-Concise (AUDIT-C)	3	~1	Adolescent girls ages 12 to 19, adult women,† pregnant women, older adults	Adolescent girls: ≥ 3 (0.96; 0.65)^32^ Adult women: ≥ 3 (0.73–0.97; 0.34–0.89)^18^ Pregnant women: > 0 (NR‡)^18^ Older adults: ≥ 4 (0.94; 0.80)^54^	Copyright: N/A Source: N/A Cost: Free online	Publicly available SAMHSA link: https://www.integration.samhsa.gov/images/res/tool_auditc.pdf
Car, Relax, Alone, Forget, Friends, Trouble (CRAFFT)^37^*	4 (additional 5 if past-year use indicated)	~2 to 3	Adolescents ages 12 to 21	≥ 1 (0.94; 0.74)^30^,^39^ Optimal cutoff score indicating heightened risk for SUD: ≥ 2 (0.79; 0.97)^39^	Copyright: 2001, Boston Children’s Hospital Source: The Center for Adolescent Substance Abuse Research, Children’s Hospital, 300 Longwood Ave., Boston, MA 02115 Phone: 617-355-5433 Email: crafft@childrens.harvard.edu Cost: N/A	Publicly available SAMHSA link which states that the CRAFFT may be reproduced in [this] exact form for use in clinical settings courtesy of the Center for Adolescent Substance Abuse Research at the Boston Children’s Hospital: https://www.integration.samhsa.gov/clinical-practice/sbirt/CRAFFT_Screening_interview.pdf Link from Boston Children’s Hospital with additional information: http://crafft.org/
NIAAA Single Item Alcohol Screening Questionnaire (SASQ)^52^	1	~1	Adults	≥ 1 (0.82; 0.79)^18^	Copyright: N/A Source: N/A Cost: N/A	Publicly available SAMHSA link to NIAAA’s *Helping Patients Who Drink Too Much: A Clinician’s Guide*, which includes NIAAA SASQ (page 4): https://www.integration.samhsa.gov/clinical-practice/Helping_Patients_Who_Drink_Too_Much.pdf Publicly available USPSTF Final Recommendation Statement: *Unhealthy Alcohol Use in Adolescents and Adults: Screening and Behavioral Counseling Interventions*, includes NIAAA SASQ question: https://www.uspreventiveservicestaskforce.org/Page/Document/RecommendationStatementFinal/unhealthy-alcohol-use-in-adolescents-and-adults-screening-and-behavioral-counseling-interventions
Quick Drinking Screen (QDS)^44^,^109^	3	~1	Adults	Scoring based on presence of NIAAA defined at-risk drinking (i.e., more than 3 drinks on any day or 7 drinks per week for adult women) in past 90 days^43^††	Copyright: 2003, Sobell & Sobell Source: Linda C. Sobell, PhD, ABPP, Center for Psychological Studies, Nova Southeastern University, 3301 College Ave., Fort Lauderdale, FL 33314 Email: sobelll@nova.edu Cost: Free	Publicly available link that states that this screener can be freely used as it is in the public domain: https://www.nova.edu/gsc/forms/quick_drinking_screen.pdf
Tolerance, Annoyed, Cut Down, Eye Opener (T-ACE)^31^	4	~1	Women of childbearing age	≥ 2 (0.69–0.88; 0.71–0.89)^25^	Copyright: 1989, Harcourt Health Sciences; permission needed to publish Sources: S. Martier, Ob/Gyn, 4707 Saint Antoine, Detroit, MI 48201 Permissions Department, Mosby, Inc. (a division of Elsevier), 6277 Sea Harbor Dr., Orlando, FL Phone: 407-345-3994 http://www.us.elsevierhealth.com/ Cost: N/A	Publicly available NIAAA link containing copyright information: https://pubs.niaaa.nih.gov/publications/t_ace.htm Publicly available NIAAA link containing T-ACE questions: https://pubs.niaaa.nih.gov/publications/arh28-2/78-79.htm
Tolerance, Worried, Eye Opener, Amnesia, K-Cut Down (TWEAK)^31^	5	~2	Pregnant women	≥ 2 (0.71–0.91; 0.73–0.83)^25^	Copyright: None Source: Marcia Russell Prevention Research Center, 1995 University Avenue, Suite 450, Berkeley, CA 94704 Phone: 510-883-5703 Email: russell@prev.org Cost: Free	Publicly available NIAAA link with more information: https://pubs.niaaa.nih.gov/publications/assessingalcohol/instrumentpdfs/74_tweak.pdf
Normal Drinker, Eye-Opener, Tolerance (NET)^47^	3	~1	Pregnant women	≥ 2 (0.61; 0.87)^47^	Copyright: 1989, Lippincott Williams & Wilkins Source: Lippincott Williams & Wilkins Permissions Department, 351 West Camden St., Baltimore, MD 21201 Phone: 410-528-4050 Email: permissions@lww.com http://www.lww.com/permissions/index.htm Cost: N/A	Not publicly available
Parents, Partner, Past, Present Pregnancy (4P’s Plus)^48^*	5	~1	Pregnant women	≥ 1 (0.87; 0.76)^48^	Copyright: The National Training Institute/NTI Upstream Source: NTI Upstream, 180 N. Michigan Ave., Suite 700, Chicago, IL 60601 Cost: Licensing fees may apply	Publicly available link with more information: https://www.ntiupstream.com/4psabout
Substance Use Brief Screen (SUBS)^53^*	4	~1	Adults	Any response other than “never” on alcohol binge question: (0.85; 0.77)	Copyright: N/A Source: N/A Cost: N/A	Publicly available NIH publication with more information: https://www.ncbi.nlm.nih.gov/pmc/articles/PMC4475501/
Cut Down, Annoyed, Guilty, Eye-Opener (CAGE)^57^	4	~1	Adults	≥ 2 (0.14–0.39; 0.97)	Copyright: None Source: N/A Cost: Freely available as it is in the public domain and no permission is necessary unless used in a profit-making endeavor	Publicly available SAMHSA link: https://www.integration.samhsa.gov/clinical-practice/sbirt/CAGE_questionaire.pdf
Michigan Alcohol Screening Test–Geriatric Version (MAST-G)^57^	24	~5 to 10	Older adults	≥ 5 (0.70–0.91; 0.81–0.85)	Copyright: 1991, The Regents of the University of Michigan Source: Frederick C. Blow, PhD, University of Michigan Alcohol Research Center, 400 E. Eisenhower Parkway, Suite A, Ann Arbor, MI 48104 Phone: 313-998-7952 Cost: Free online	Publicly available NIH link to SAMHSA’s *Substance Abuse Among Older Adults: Treatment Improvement Protocol No. 26* (page 55): https://www.ncbi.nlm.nih.gov/books/NBK64419/pdf/Bookshelf_NBK64419.pdf
Short Michigan Alcohol Screening Test–Geriatric Version (SMAST-G)^57^	10	Not reported	Older adults	≥ 2 (0.52; 0.96)	Copyright: 1991, The Regents of the University of Michigan Source: N/A Cost: N/A	Publicly available link provided by The Hartford Institute for Geriatric Nursing, New York University, Rory Meyers College of Nursing: https://consultgeri.org/try-this/general-assessment/issue-17.pdf
Comorbidity Alcohol Risk Evaluation Tool (CARET)	10	~2 to 5	Older adults	A positive response in any of the seven risk categories (0.92; 0.51)^54^	Copyright: N/A Source: N/A Cost: N/A	Not publicly available

NIAAA = National Institute on Alcohol Abuse and Alcoholism; NIDA = National Institute on Drug Abuse; NIH = National Institutes of Health; SAMHSA = Substance Abuse and Mental Health Services Administration.

* Instrument screens for alcohol and other substances.

† Recommended AUDIT-C cutoff score is different for adult women (≥ 3) and men (≥ 4).^18^

‡ Not reported.

§ Recommended AUDIT cutoff score is the same for adult women and men (≥ 8).^18^

** Several U.S.-based studies show more optimal balances of sensitivity and specificity at lower AUDIT cutoffs (e.g., 3, 4, 5); preliminary findings from the USPSTF 2018 updated evidence report and systematic review indicates that lower cutoffs may be preferred.^18^

†† Sensitivity and specificity are not reported for this instrument.

‡‡ N/A, information was not available or retrievable. None, the instrument explicitly states that no copyright is held. Cost: N/A, no information was found regarding cost. Free/free online, the information pertaining to the instrument explicitly states that it is available to the public.
